# Spatio-temporal dynamics of sulfur bacteria during oxic--anoxic regime shifts in a seasonally stratified lake

**DOI:** 10.1093/femsec/fiy040

**Published:** 2018-03-08

**Authors:** Muhe Diao, Jef Huisman, Gerard Muyzer

**Affiliations:** Department of Freshwater and Marine Ecology, Institute for Biodiversity and Ecosystem Dynamics, University of Amsterdam, 1090 GE Amsterdam, The Netherlands

**Keywords:** Sulfur-oxidizing bacteria, sulfate-reducing bacteria, green sulfur bacteria, purple sulfur bacteria, regime shifts, stratified lakes

## Abstract

Sulfate-reducing bacteria (SRB) and sulfur-oxidizing bacteria drive major transformations in the sulfur cycle, and play vital roles in oxic--anoxic transitions in lakes and coastal waters. However, information on the succession of these sulfur bacteria in seasonally stratified lakes using molecular biological techniques is scarce. Here, we used 16S rRNA gene amplicon sequencing to study the spatio-temporal dynamics of sulfur bacteria during oxic--anoxic regime shifts in Lake Vechten. Oxygen and sulfate were mixed throughout the water column in winter and early spring. Meanwhile, SRB, green sulfur bacteria (GSB), purple sulfur bacteria (PSB), and colorless sulfur bacteria (CSB) exclusively inhabited the sediment. After the water column stratified, oxygen and nitrate concentrations decreased in the hypolimnion and various SRB species expanded into the anoxic hypolimnion. Consequently, sulfate was reduced to sulfide, stimulating the growth of PSB and GSB in the metalimnion and hypolimnion during summer stratification. When hypoxia spread throughout the water column during fall turnover, SRB and GSB vanished from the water column, whereas CSB (mainly *Arcobacter*) and PSB (*Lamprocystis*) became dominant and oxidized the accumulated sulfide under micro-aerobic conditions. Our results support the view that, once ecosystems have become anoxic and sulfidic, a large oxygen influx is needed to overcome the anaerobic sulfur cycle and bring the ecosystems back into their oxic state.

## Teaser

The succession of sulfur bacteria during oxic--anoxic transitions in a seasonally stratified lake was investigated and summarized in a conceptual model.

## INTRODUCTION

Oxygen depletion may lead to hypoxia and anoxia in lakes, coastal waters and the open ocean, which is detrimental to many aquatic organisms (Diaz and Rosenberg 1995; Vaquer-Sunyer and Duarte 2008; Breitburg *et al*. 2018). During the past decades, the frequency, intensity and duration of hypoxia and anoxia seem to have increased due to combined effects of eutrophication and global warming, resulting in an expanding area of ‘dead zones’ in eutrophied waters across the globe (Diaz and Rosenberg 2008; Middelburg and Levin 2009; Jenny *et al*. 2016). The transition from oxic to anoxic water is accompanied by major changes in microbial community structure and biogeochemical processes. However, a detailed understanding of the different biological and chemical feedbacks involved in oxic--anoxic transitions is still lacking.

Recently, Bush *et al*. (2017) developed a mathematical model to investigate interactions between microbial community composition and the dissolved oxygen (DO) concentration. The model predicts that the transition from oxic to anoxic water may occur in the form of a regime shift. Regime shifts are defined as abrupt, large and persistent changes in the structure and function of ecosystems triggered by gradual changes in environmental conditions (Chavez *et al*. 2003; Scheffer and Carpenter 2003; Biggs, Carpenter and Brock 2009). When the oxygen influx is gradually reduced, at first oxygen-producing cyanobacteria and algae still persist and the lake remains in an oxic state. Once the oxygen concentration is suppressed below a critical threshold, sulfate-reducing bacteria (SRB) and photosynthetic sulfur bacteria take over. The SRB cause an increase in sulfide concentration, which suppresses cyanobacterial growth, and hence the lake rapidly shifts from an oxic into an anoxic state. One of the implications predicted by the model is that this oxic--anoxic regime shift displays hysteresis. Once the water has turned anoxic, high sulfide concentrations maintained by SRB stabilize the anoxic conditions. As a consequence, a large oxygen influx is needed to bring the system back into its oxic state. These model predictions are supported by observations from a seasonally stratified lake, which shows hysteresis in the transition between oxic and anoxic states with similar changes in microbial community composition as predicted by the model (Bush *et al*. 2017). Hysteresis effects have also been reported for anoxia in coastal ecosystems (e.g. Conley *et al*. 2009; Middelburg and Levin 2009; Zhang *et al*. 2010).

SRB and sulfur-oxidizing bacteria (SOB) thus play important roles in oxic--anoxic regime shifts, as they drive major transformations in the sulfur cycle (Muyzer and Stams 2008; Frigaard and Dahl 2009; Ghosh and Dam 2009; Muyzer, Kuenen and Robertson 2013). SRB use sulfate as electron acceptor to degrade organic compounds, producing sulfide as end product (Muyzer and Stams 2008). SOB comprise several groups, including colorless sulfur bacteria (CSB), green sulfur bacteria (GSB) and purple sulfur bacteria (PSB). CSB oxidize reduced sulfur compounds with oxygen or nitrate as electron acceptor (Sweerts *et al*. 1990; Muyzer, Kuenen and Robertson 2013). GSB and PSB can use sulfide, elemental sulfur and thiosulfate as electron donors in anoxygenic photosynthesis (Pfennig and Trüper 1989; Frigaard and Dahl 2009; Gregersen, Bryant and Frigaard 2011).

In the past, several excellent attempts at an overarching appraisal of sulfuretum communities have been made (Guerrero *et al*. 1985; Camacho, Vicente and Miracle 2000; Tonolla *et al*. 2003), but these studies were to some extent hampered by the inherent limitations of classic microscopic observations. With the advancement of modern molecular-genetic techniques, a much deeper understanding of the dynamics, structure and functioning of microbial communities can be obtained. Yet, although some bacterial groups have been studied in detail (Edwardson and Hollibaugh 2017; Llorens-Marès *et al*. 2017), comprehensive studies of the overall community dynamics of sulfur bacteria in stratified lakes have received less attention in recent years. For instance, Bush *et al*. (2017) provided data on the seasonal succession of cyanobacteria, phototrophic sulfur bacteria and SRB in the metalimnion, but a detailed analysis of the sulfur bacteria at a higher taxonomic resolution and across the entire depth range of the lake is lacking. Other studies have analyzed the overall composition of lake bacteria at a coarse taxonomic resolution, but do not specifically zoom in at the spatio-temporal dynamics of the different genera involved in the sulfur cycle (Eiler, Heinrich and Bertilsson 2012; Diao *et al*. 2017). As a consequence, the diversity, abundance and seasonal dynamics of sulfur bacteria during oxic--anoxic transitions in seasonally stratified lakes are not yet well understood.

Here, we investigated the spatio-temporal dynamics of different functional groups of sulfur bacteria (including SRB, GSB, PSB and CSB) in Lake Vechten, a seasonally stratified lake that is well mixed during winter, while it develops an anoxic and sulfidic hypolimnion during summer stratification (Steenbergen and Korthals 1982; Steenbergen and Verdouw 1982; Bush *et al*. 2017). Specific objectives of our study were (i) to determine the taxonomic composition, at the genus level, of the SRB, GSB, PSB and CSB in this seasonally stratified lake, (ii) to determine seasonal changes in their diversity, distribution and relative abundances, and (iii) to investigate how their successional trajectories are related to seasonal changes between oxic and anoxic conditions. For this purpose, samples from different water layers were collected monthly over a period of 1 year, and 16S rRNA gene amplicon sequencing was applied to identify the diversity and composition of the sulfur bacteria. These results were correlated to seasonal changes in environmental parameters.

## MATERIALS AND METHODS

### Lake, sampling and general analysis

Lake Vechten (52°04’N, 5°05’E) is a small eutrophic lake near the city of Utrecht, in the center of The Netherlands. It consists of two connected basins with a total surface area of 4.7 ha, and has a maximum depth of 11.9 m (Steenbergen and Verdouw 1982). Temperature, DO, photosynthetically active radiation (PAR) and pH of lake water were measured with a multiprobe Hydrolab DataSonde 4a (Hydrolab Corporation, Austin, TX, USA). Water samples were collected biweekly to monthly from every meter in the western basin from March 2013 to April 2014. Water was pumped via a hose connected to the Hydrolab to make sure the samples matched the conditions measured at that particular depth. The samples were filtered through 0.20 µm nylon membrane filters (Millipore, GNWP) to collect bacterial cells. The filters were frozen immediately and preserved in a −20°C freezer until further processing. Sediment samples (0–10 cm) were collected monthly with a box-corer from the same location, transported to the laboratory in a dry shipper and stored at −20°C until further analysis. Dry weight of sediment was determined after drying for 2 days in a 60°C oven.

Concentrations of sulfate (SO_4_^2−^), ammonium (NH_4_^+^) and nitrate (NO_3_^−^) in the filtered water samples were measured by an auto-analyzer (SAN^++^, Skalar, The Netherlands). For sulfide, lake water was filtered through 0.20 µm polyethersulfone membrane filters and fixed with zinc acetate (at a final concentration of 2% w/v) in the field. Afterwards, sulfide was measured in the laboratory according to the methylene blue method (Trüper and Schlegel 1964). Dissolved organic carbon (DOC) was measured by a total organic carbon analyzer (TOC-V_CPH_, Shimadzu, Japan). The data were visualized with Ocean Data View 4.7.8 (Schlitzer 2002).

### DNA extraction

DNA was extracted from the collected bacterial cells using the PowerSoil DNA Isolation Kit according to the manufacturer's instructions (Mo Bio, Laboratories Inc., USA). The concentration of extracted DNA was quantified with the Qubit dsDNA BR Assay Kit (Invitrogen, USA).

### Amplicon sequencing and operational taxonomic unit assignments

The PCR-amplified 16S rRNA genes of 189 water samples and 11 sediment samples were profiled by denaturing gradient gel electrophoresis (DGGE). Based on the DGGE profiles and measured vertical stratification pattern of Lake Vechten, we selected 51 samples from the water column and 4 samples from the sediment for 16S rRNA gene amplicon sequencing ( Table S1, Supporting Information). Sequencing was performed on an Illumina MiSeq system by Research and Testing Laboratory (Lubbock, Texas, USA). The primer pair S-D-Bact-0341-b-S-17 (5΄-CCTACGGGNGGCWGCAG-3΄) and S-D-Bact-0785-a-A-21 (5΄-GACTACHVGGGTATCTAATCC-3΄) was used to generate paired-end sequence reads, covering the V3-V4 region of the 16S rRNA gene (Herlemann *et al*. 2011).

The forward and reverse reads were taken in FASTQ format and were merged using the PEAR Illumina paired-end read merger (Zhang *et al*. 2014). Reads were run through an internally developed quality trimming algorithm. Prefix dereplication was performed using the USEARCH dereplication algorithm (Edgar 2010). Clustering at a 4% divergence was applied using the USEARCH clustering algorithm (Edgar 2010). Operational taxonomic units (OTUs, 97% similarity) were selected using the UPARSE OTU selection algorithm (Edgar 2013). Chimera checking was performed on the selected OTUs using the UCHIME chimera detection software executed in *de novo* mode (Edgar *et al*. 2011). All chimeric sequences were removed and the corrected sequences were then written to the output file. After quality filtering a total of 2934 111 sequences were obtained with an average sequence length of 420 bp.

The sequences were run through the USEARCH global alignment program along with a python program to determine the taxonomic information for each sequence (Bokulich *et al*. 2015), using a database of high-quality sequences derived from the NCBI database up to May 2014. From the top 6 sequence matches, a confidence value was estimated at each taxonomic level (phylum, class, order, family, genus and species). Once confidence values were assigned for each sequence, an RDP-formatted output file was generated for the final analysis in USEARCH. Subsequently, the data were entered into the diversity analysis program that takes the OTU/dereplication table output from sequence clustering along with the output generated during taxonomic identification and generates a new OTU table with the taxonomic information tied to each cluster.

The 16S amplicon sequences have been deposited as dataset SAMN06314865-SAMN06314918 in the Sequence Read Archive (SRA, NCBI).

### Statistical analysis

We applied redundancy analysis (RDA; Zuur *et al*. 2009; Oksanen *et al*. 2013) to investigate associations between environmental parameters and the taxonomic composition of the sulfur bacteria. To avoid data with many zeroes, we selected only those bacterial genera that were present in at least 15 water samples. The analysis was performed using R (version 3.0.3), with the measured environmental parameters as explanatory variables and the 16S-based relative abundances of the bacterial genera as response variables. All environmental parameters, except pH, were log (x + 1)-transferred prior to the analysis. Subsequently, the number of explanatory variables was reduced by stepwise removal of variables with a high variance inflation factor (VIF) using the R function VIF in the *car* package (Fox and Weisberg 2011), until only variables with a VIF <10 remained. Finally, RDA was applied using forward selection with the Ordistep function in the R package *vegan* to select those explanatory variables that contributed significantly to the RDA model, while removing nonsignificant terms (Oksanen *et al*. 2013). Significance was based on a permutation test using the multivariate pseudo-F statistic and 9999 permutations (Zuur *et al*. 2009).

## RESULTS

### Environmental conditions

**Figure 1.**
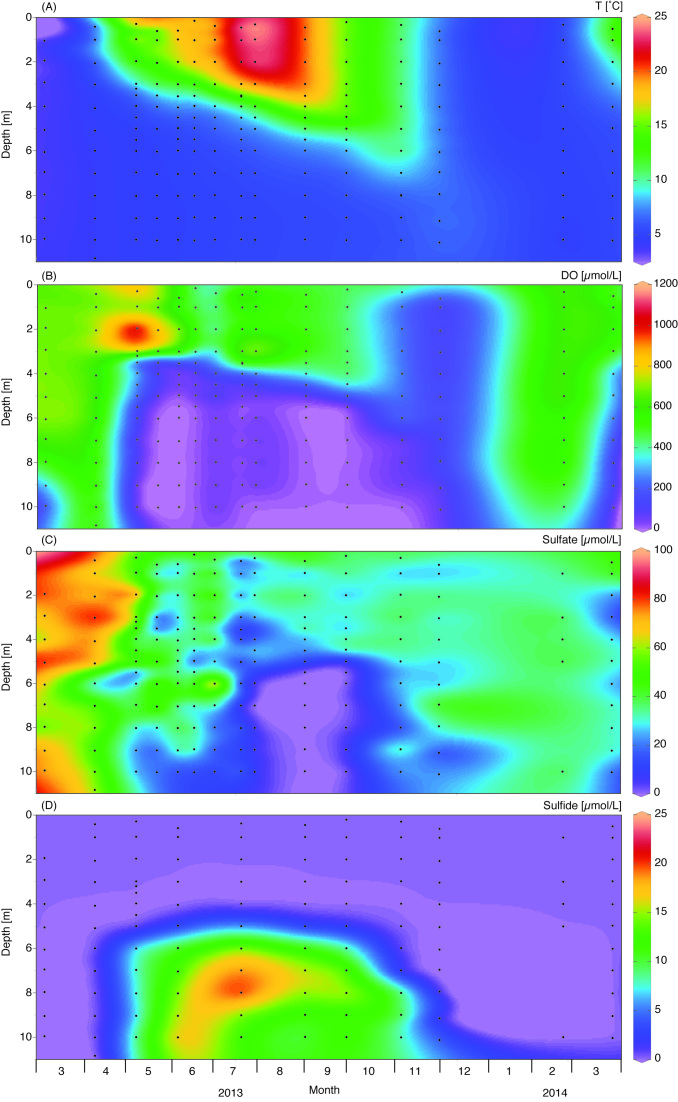
Spatio-temporal dynamics of environmental variables in Lake Vechten. (A) Temperature; (B) dissolved oxygen; (C) sulfate; (D) sulfide.

The temperature in Lake Vechten was almost uniform over the entire depth in early spring, and the water column was saturated with oxygen (Fig. 1A and B). When the surface temperature increased in April, the water column stratified. The epilimnion remained oxic, whereas the hypolimnion became anoxic during summer stratification. The stratification persisted until the water column was mixed during fall turnover. The oxygen concentration in the surface layer decreased during fall turnover, creating hypoxia throughout the water column in November. This was followed by re-oxygenation of the water column during the winter period (Fig. 1B).

The sulfate concentration was 60–90 μM throughout the water column in early spring, while the sulfide concentration was negligible during this period (Fig. 1C and D). When the lake stratified, the sulfate concentration in the anoxic hypolimnion decreased to less than 10 μM, whereas sulfide increased to 10–20 μM. Interestingly, the relatively high sulfide concentration of the hypolimnion did not expand into the surface layer when hypoxia spread throughout the water column during fall turnover. Instead, sulfide vanished during fall turnover, while the sulfate concentration increased to 30–50 µM (Fig. 1D).

The nitrate concentration was relatively high in early spring, was depleted throughout the entire water column from May until December, and increased again when the lake was mixed during the winter period (Fig. S1A, Supporting Information). Conversely, both ammonium and DOC had low concentrations in the winter period, but accumulated in the anoxic hypolimnion during summer stratification (Fig. S1B and S1C, Supporting Information).

**Figure 2.**
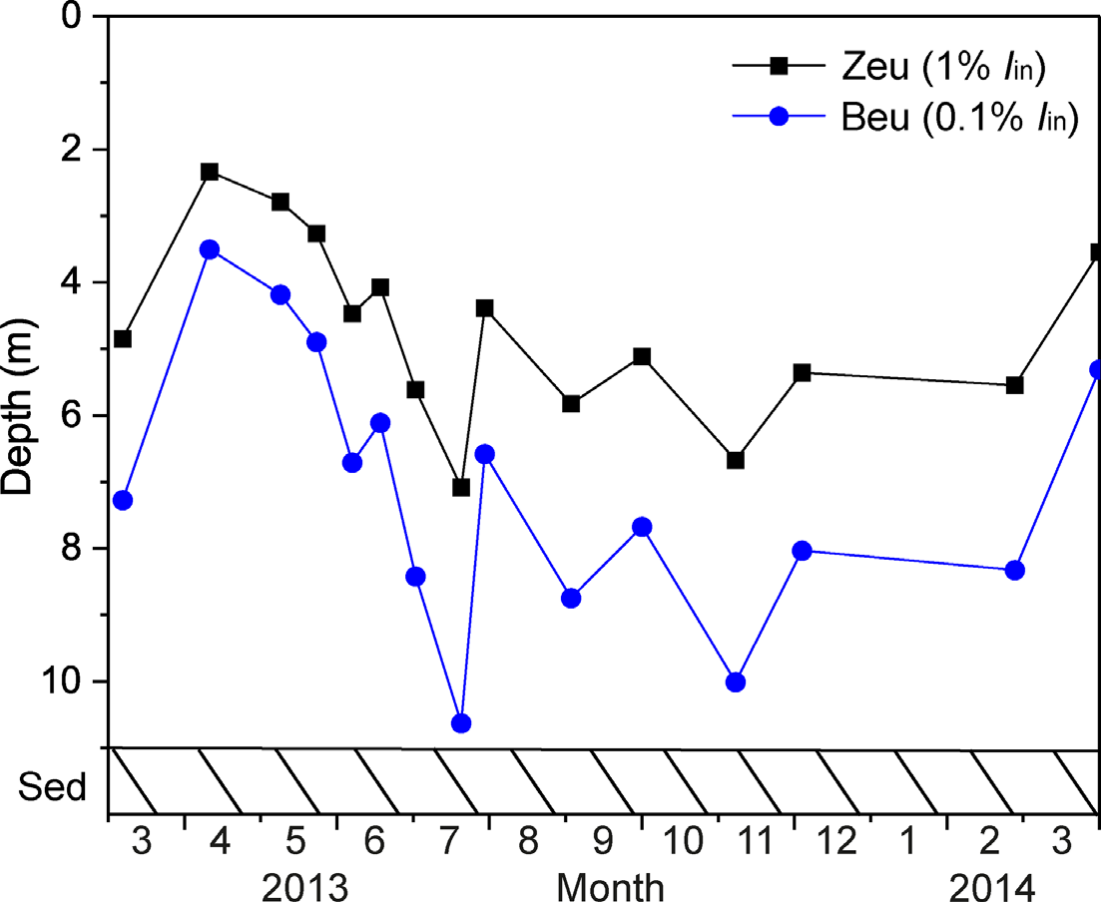
Dynamics of euphotic depth (Z_eu_) and bacterial euphotic depth (B_eu_) in Lake Vechten.

To investigate light penetration in Lake Vechten, we measured the euphotic depth (i.e. the depth at which irradiance in the PAR range is 1% of the surface irradiance), which indicates the maximum depth of the light zone suitable for oxygenic photosynthesis by cyanobacteria and eukaryotic phytoplankton. Because anoxygenic phototrophs (in particular GSB) can grow at lower light levels than phytoplankton species (Overmann, Cypionka and Pfennig 1992; Manske *et al*. 2005), we introduce the term ‘bacterial euphotic depth’ (which we define as the depth at which the irradiance is 0.1% of the surface irradiance). Light penetrated quite deep into the water column in March 2013, but the euphotic depth and bacterial euphotic depth shifted upwards to <2.5 m and <3.5 m, respectively, during the phytoplankton spring bloom in April (Fig. 2). When the phytoplankton bloom in the surface layer declined in June, presumably because of nutrient limitation (Diao *et al*. 2017), light could enter into the deeper water layers again and bacterial euphotic depth reached 10.6 m in July. In the subsequent spring, the euphotic depth and bacterial euphotic depth shallowed again during the phytoplankton spring bloom.

### Microbial community composition

Amplicon sequencing of bacterial 16S rRNA genes showed that SRB were represented by *Deltaproteobacteria*, including members of the families *Desulfobulbaceae*, *Desulfovibrionaceae*, *Desulfobacteraceae*, *Desulfuromonadaceae* and *Syntrophaceae* (Table 1). *Desulfobulbus* was the most abundant SRB genus in the water column, while *Desulfobacterium* was the dominant SRB genus in the sediment. GSB belonged to the family *Chlorobiaceae* of the phylum *Chlorobi*, while PSB belonged to the family *Chromatiaceae* of the class *Gammaproteobacteria*. CSB in the water column were mainly from the class *Epsilonproteobacteria* (with *Arcobacter* as the most abundant genus), whereas CSB in the sediment were dominated by *Betaproteobacteria* (mostly *Thiobacillus*). In total, the number of genera belonging to the sulfur bacteria was higher in the water column than in the sediment.

**Table 1.** Diversity of sulfur bacteria in Lake Vechten.^a^

			Genus	
Group	Phylum/Class	Family	Water column	Sediment
SRB	*Deltaproteobacteria*	*Desulfobulbaceae*	*Desulfobulbus*	*Desulfobulbus*
			unknown	n.d.
		*Desulfovibrionaceae*	*Desulfovibrio*	n.d.
		*Desulfobacteraceae*	*Desulfobacterium*	*Desulfobacterium*
			n.d.	unknown
		*Desulfuromonadaceae*	*n.d*.	*Desulfuromonas*
		*Syntrophaceae*	*Desulfomonile*	n.d.
	*Epsilonproteobacteria*	*Campylobacteraceae*	*Sulfurospirillum* ^b^	n.d.
GSB	*Chlorobi*	*Chlorobiaceae*	unknown	n.d.
			*Chlorobium*	*Chlorobium*
PSB	*Gammaproteobacteria*	*Chromatiaceae*	*Thiodictyon*	*Thiodictyon*
			*Lamprocystis*	*Lamprocystis*
			*Thiocystis*	n.d.
			*Thiorhodococcus*	n.d.
CSB	*Epsilonproteobacteria*	*Campylobacteraceae*	*Arcobacter*	n.d.
			*Sulfurospirillum* ^b^	n.d.
		*Helicobacteraceae*	*Sulfurimonas*	n.d.
	*Betaproteobacteria*	*Hydrogenophilaceae*	n.d.	*Thiobacillus*

^a^Only genera representing >0.1% of the total bacterial community in at least one of the samples are shown; n.d. = not detected.

^b^ *Sulfurospirillum* can reduce elemental sulfur (Sorokin, Tourova and Muyzer 2013) and it can also oxidize sulfide with nitrate (Eisenmann *et al*. 1995). Therefore, it is listed in both the SRB and CSB.

**Figure 3.**
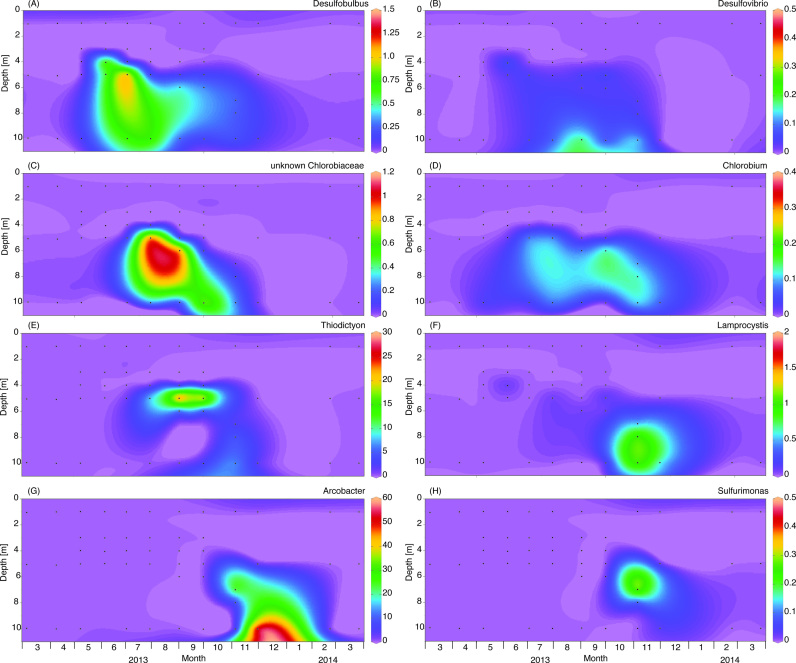
Spatio-temporal dynamics of sulfur bacteria (genus level) in the water column of Lake Vechten, based on 16S rRNA gene amplicon sequences. The graphs show the relative abundances (%) and distributions of the sulfate-reducing bacteria (A) *Desulfobulbus* and (B) *Desulfovibrio*, the green sulfur bacteria (C) unknown *Chlorobiaceae* and (D) *Chlorobium*, the purple sulfur bacteria (E) *Thiodictyon* and (F) *Lamprocystis*, and the colorless sulfur bacteria (G) *Arcobacter* and (H) *Sulfurimonas*.

Spatio-temporal dynamics of the most abundant genera of sulfur bacteria are shown in Fig. 3. Members belonging to the SRB genera *Desulfobulbus* and *Desulfovibrio* were present in the metalimnion and hypolimnion when the lake became stratified (Fig. 3A and B). Similar distributions were observed for the SRB genera *Desulfobacterium* and *Desulfomonile* (Fig. S2, Supporting Information). GSB of the genus *Chlorobium* and other unknown genera were present in the metalimnion and hypolimnion only when the lake was stratified (Fig. 3C and D). PSB of the genus *Thiodictyon* reached high relative abundances in the metalimnion (Fig. 3E), where it accounted for more than 20% of the total bacterial community in late summer. Furthermore, *Thiocystis* was found in the hypolimnion in late spring, *Thiorhodococcus* in the metalimnion in the summer and hypolimnion in the fall, and *Lamprocystis* in the hypolimnion in the fall (Fig. 3F and Fig. S3, Supporting Information). The CSB genera *Arcobacter* and *Sulfurimonas* were prevalent at 7–10 m depth in November and December (Fig. 3G and H). Especially *Arcobacter* was highly abundant, constituting almost 60% of the total bacterial community in the bottom water layer in December.

**Figure 4.**
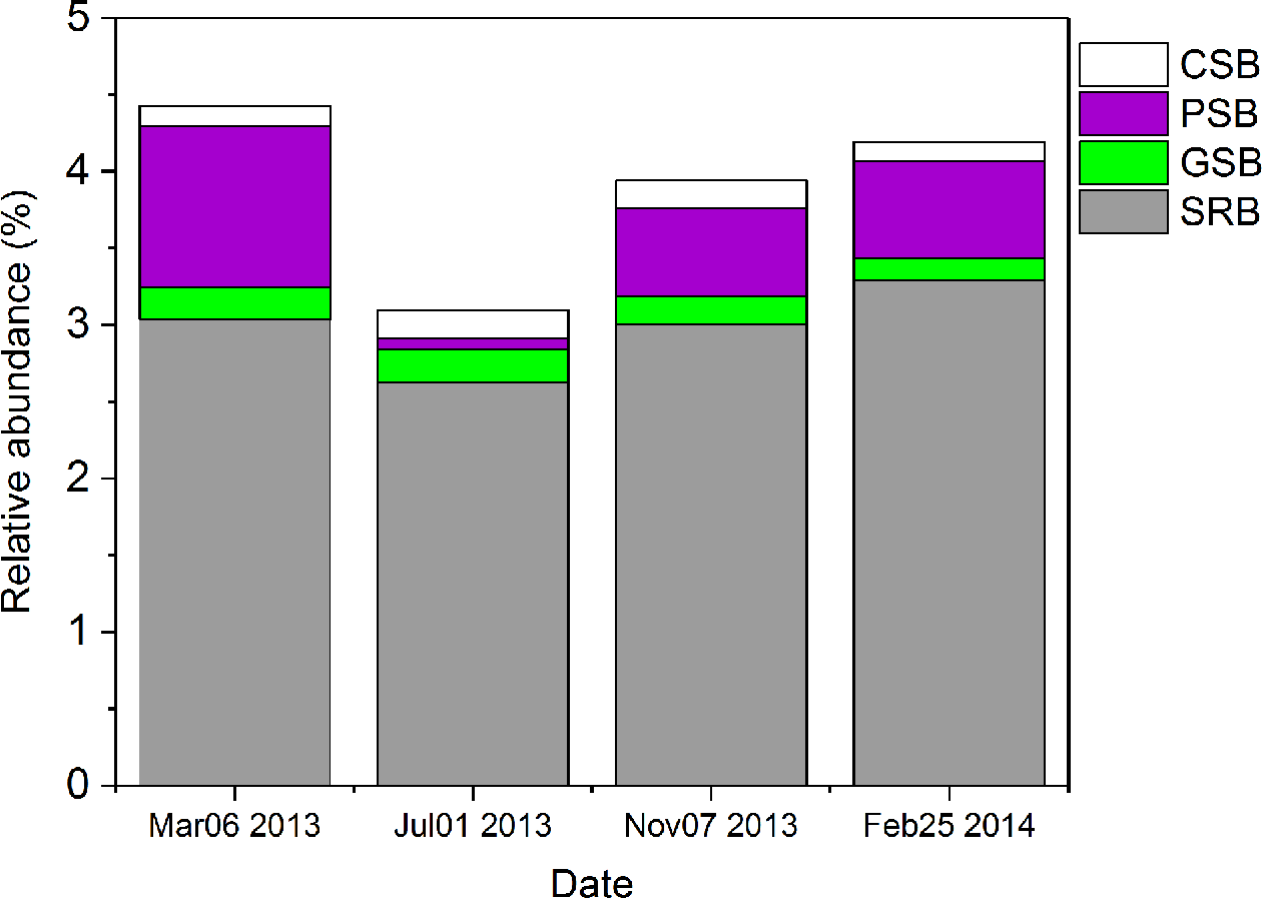
Spatio-temporal dynamics of the relative abundances of sulfur bacteria in the sediment of Lake Vechten, based on 16S rRNA gene amplicon sequences.

In the sediment, SRB were by far the most abundant sulfur bacteria throughout the seasons (Fig. 4). The relative abundance of PSB was quite high in spring, decreased sharply during summer stratification, and increased again after fall turnover. There were no clear seasonal changes in the relative abundances of GSB and CSB in the sediment (Fig. 4).

### Linking sulfur bacteria with environmental drivers

Redundancy analysis was applied to correlate the relative abundances of sulfur bacteria (at genus level) in the water column with environmental variables. In total nine explanatory variables had a VIF <10, including temperature, DO, PAR, NH_4_^+^, NO_3_^−^, PO_4_^3−^, SO_4_^2−^, DOC and sulfide. Forward selection revealed that four of these nine variables were significant in the redundancy analysis: sulfide, DO, SO_4_^2−^ and NH_4_^+^ (Table S2, Supporting Information).

**Figure 5.**
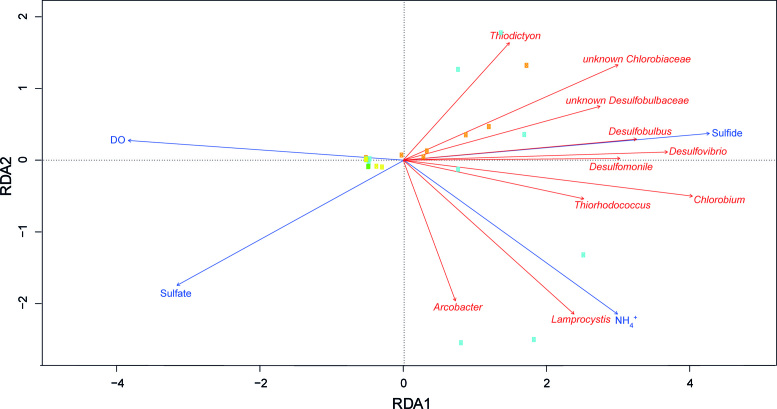
Redundancy analysis of the effect of environmental variables on the taxonomic composition of sulfur bacteria in the water column. The response variables (red arrows) were the relative abundances of sulfur bacteria. To avoid many zeroes in the data, we used only genera that were present in at least 15 water samples. Squares represent the samping points (yellow = spring; orange = summer; cyan = fall; green = winter). Total variation explained by the RDA model was 35.1%.

The first and second axis of the RDA plot explained 27.5% and 4.8% of the variation in the composition of sulfur bacteria, respectively (Fig. 5). Relative abundances of SRB (*Desulfobulbus*, *Desulfovibrio*, *Desulfomonile*, unknown *Desulfobulbacaea*) and GSB (*Chlorobium*, unknown *Chlorobiaceae*) were positively correlated with the sulfide concentration, and negatively correlated with DO. Furthermore, some PSB (*Thiodictyon*), GSB (unknown *Chlorobiaceae*) and SRB (unknown *Desulfobulbacaea*) were negatively correlated with the sulfate concentration, whereas other PSB (*Lamprocystis*) were positively correlated with the ammonium concentration.

## DISCUSSION

Our results show that Lake Vechten is characterized by distinct seasonal changes in the oxygen, sulfate and sulfide concentrations and a marked seasonal succession of a wide diversity of sulfur bacteria. For a proper interpretation of these results, it is important to realize that sulfur bacteria do not only track changes in oxygen and sulfur availability, but are also important drivers of these changes as they play key roles in oxidation--reduction reactions of the sulfur cycle. We will first discuss our observations on the taxonomic composition and dynamics of different functional groups of sulfur bacteria, and then summarize these results into a conceptual model of the seasonal succession of sulfur bacteria during oxic--anoxic regime shifts in seasonally stratified lakes.

### Sulfate-reducing bacteria

SRB are anaerobic bacteria that oxidize organic matter (and hydrogen) using sulfate as terminal electron acceptor (Muyzer and Stams 2008). During seasonal succession in Lake Vechten, SRB were preceded by denitrifying bacteria, which reached peak abundances in the hypolimnion at the onset of lake stratification in May (data not shown). Sulfate reduction has a lower energy yield than nitrate reduction. Hence, as expected, SRB replaced the denitrifying bacteria in the hypolimnion and metalimnion of the lake during the summer months, after nitrate had been depleted. SRB produced sulfide, and their presence coincided with the observed decline of the sulfate concentration and accumulation of sulfide in the hypolimnion of the lake.

Based on bacterial 16S rRNA gene amplicon sequencing, *Desulfobulbus*, *Desulfovibrio*, *Desulfobacterium*, *Desulfomonile* and *Desulfuromonas* were the dominant SRB genera in the metalimnion and hypolimnion of Lake Vechten, which was also found in previous studies for other freshwater lakes (Kondo *et al*. 2006; Kubo, Kojima and Fukui 2014).

Although we did not detect SRB in the water column in winter, SRB were present in the sediment during the winter period, when an influx of sulfate from the water column into the sediment may have stimulated their activity. *Desulfobulbus* and *Desulfobacterium* were found in the sediment throughout the year and in the anoxic hypolimnion during summer. Hence, most likely the sediment acted as a seed bank (*sensu* Lennon and Jones 2011) for these SRB, which initiated the succession of SRB in the anoxic hypolimnion during summer stratification.

### Green sulfur bacteria

GSB are commonly found in the anoxic water layers of stratified lakes and marine ecosystems (Mori *et al*. 2013; Saarenheimo *et al*. 2016). They are often found below a layer of PSB, presumably because GSB are better adapted to very low light levels than PSB (Biebl and Pfennig 1978; Guerrero *et al*. 1985; Vila and Abella 1994). For instance, a strain isolated from the chemocline of the Black Sea, at 100 m depth, could still photosynthesize at 0.015 µmol quanta m^−2^ s^−1^ and its photosynthetic rate reached light saturation at 1 µmol quanta m^−2^ s^−1^ (Overmann, Cypionka and Pfennig 1992; Manske *et al*. 2005), which is 0.05%–0.10% of the surface irradiance on a sunny day. Furthermore, GSB are obligate anaerobes, whereas many PSB can tolerate and sometimes even utilize oxygen. Hence, the anoxic hypolimnion of Lake Vechten provided suitable conditions for GSB during the summer period. Interestingly, GSB reached peak abundances in the hypolimnion from July onwards (Fig. 3C and D). At this time, light levels reaching the hypolimnion may have been sufficient for GSB photosynthesis, just after the development of the phytoplankton spring bloom that prevented light from reaching the hypolimnion in April and May (Fig. 2).

The GSB in our study showed a similar spatio-temporal distribution as the SRB. This may indicate that sulfide produced by SRB stimulated the proliferation of GSB, as has been observed in mixed laboratory cultures (Biebl and Pfennig 1978). Conversely, the photosynthetic activity of GSB and PSB produces oxidized sulfur species that can be utilized as terminal electron acceptor by SRB. This mutual reciprocity may result in a tight sulfur cycle in which sulfur is shuttled back and forth between the different functional groups, fueled by the photosynthetic activity of GSB and PSB and the degradation of organic matter by SRB (Hamilton *et al*. 2014; Bush *et al*. 2017).

### Purple sulfur bacteria

Bacterial 16S rRNA sequencing data revealed four different PSB genera in the meta- and hypolimnion of Lake Vechten. *Thiocystis* peaked just above the sediment in early May, soon after the onset of lake stratification. At that time, a dense phytoplankton spring bloom absorbed most of the incident light, and although the spectral distribution of the underwater light field was not measured, light penetration to 10 m depth was less than 0.1 µmol photons m^−2^ s^−1^ in the PAR range (cf. Fig. 2), which seems too low to support photoautotrophic growth of PSB. However, it is known that several PSB are not obligate photoautotrophs, but can also grow in the dark as chemolithoautotrophs oxidizing reduced sulfur compounds under micro-aerobic and sometimes even oxic conditions (Schaub and van Gemerden 1994; Camacho, Vicente and Miracle 2000; Casamayor, García-Cantizano and Pedrós-Alió 2008). Chemolithoautotrophic and chemoheterotrophic growth has been reported for several *Thiocystis* spp. (Kämpf and Pfennig 1980; Peduzzi *et al*. 2011), and offers a plausible explanation for the peak abundance of *Thiocystis* just above the sediment when the lake started to develop a sulfidic hypolimnion.

*Thiodictyon* and to a lesser extent also *Thiorhodococcus* reached high relative abundances in the metalimnion during the summer months. Light levels reaching the metalimnion in the period July--September were roughly ∼1% of the surface light intensity, providing suitable light conditions for photoautotrophic growth of PSB. *Thiodictyon* cells are non-motile, but often form aggregates and contain large gas vesicles (Cohen-Bazire, Kunisawa and Pfennig 1969; Walsby 1994). Hence, *Thiodictyon* probably reached high relative abundances in the metalimnion through buoyancy regulation provided by its gas vesicles. *Thiorhodococcus* is a motile bacterium (Guyoneaud, Caumette and Imhoff 2015), which may have enabled its aggregation in the metalimnion.

*Lamprocystis* became dominant in the deeper parts of the lake in November. It is possible that it could benefit from the very low light levels at these depths, but some *Lamprocystis* species can also grow in the dark as chemolithoautotrophs under micro-aerobic conditions (Imhoff 2001). Hence, *Lamprocystis* may have contributed to the chemotrophic oxidation of sulfide when deep mixing brought low oxygen levels into the former hypolimnion during fall turnover.

### Colorless sulfur bacteria

CSB are key players in the oxidation of reduced sulfur compounds, and often thrive under micro-aerobic and aerobic conditions. In Lake Vechten they were represented by *Epsilonpreoteobacteria* and *Betaproteobacteria*, similar to several other freshwater lakes (Biderre-Petit *et al*. 2011; Yang *et al*. 2013; Hamilton *et al*. 2014). The CSB genera *Arcobacter* and *Sulfurimonas* became abundant in deep water layers during the fall, where they most likely were involved in oxidation of the accumulated sulfide using the low oxygen levels that were mixed into the former hypolimnion during fall turnover. *Arcobacter* is a highly motile bacterium that can form swarms at the oxic--anoxic interface, and is able to grow at very low oxygen concentrations in comparison to other CSB (Sievert *et al*. 2007). Similar seasonal shifts from phototrophic sulfur bacteria to CSB have been reported in the chemocline of a meromictic lake in winter (Noguerola *et al*. 2015) and in a seasonally stratified marine lake after fall turnover (Pjevac *et al*. 2015), most likely because the increasing oxygen levels suppress GSB and to a lesser extent also PSB while favoring the oxidation activities of CSB.

Previous studies have reported that sulfur-oxidizing *Beggiatoa* were abundant in the mats on the sediment of Lake Vechten (Sweerts *et al*. 1990), but we have not detected *Beggiatoa* or close relatives in the water column or sediment of Lake Vechten. Instead, *Thiobacillus* was the dominant CSB in the sediment, particularly during the winter period.

### Seasonal succession of sulfur bacteria

**Figure 6.**
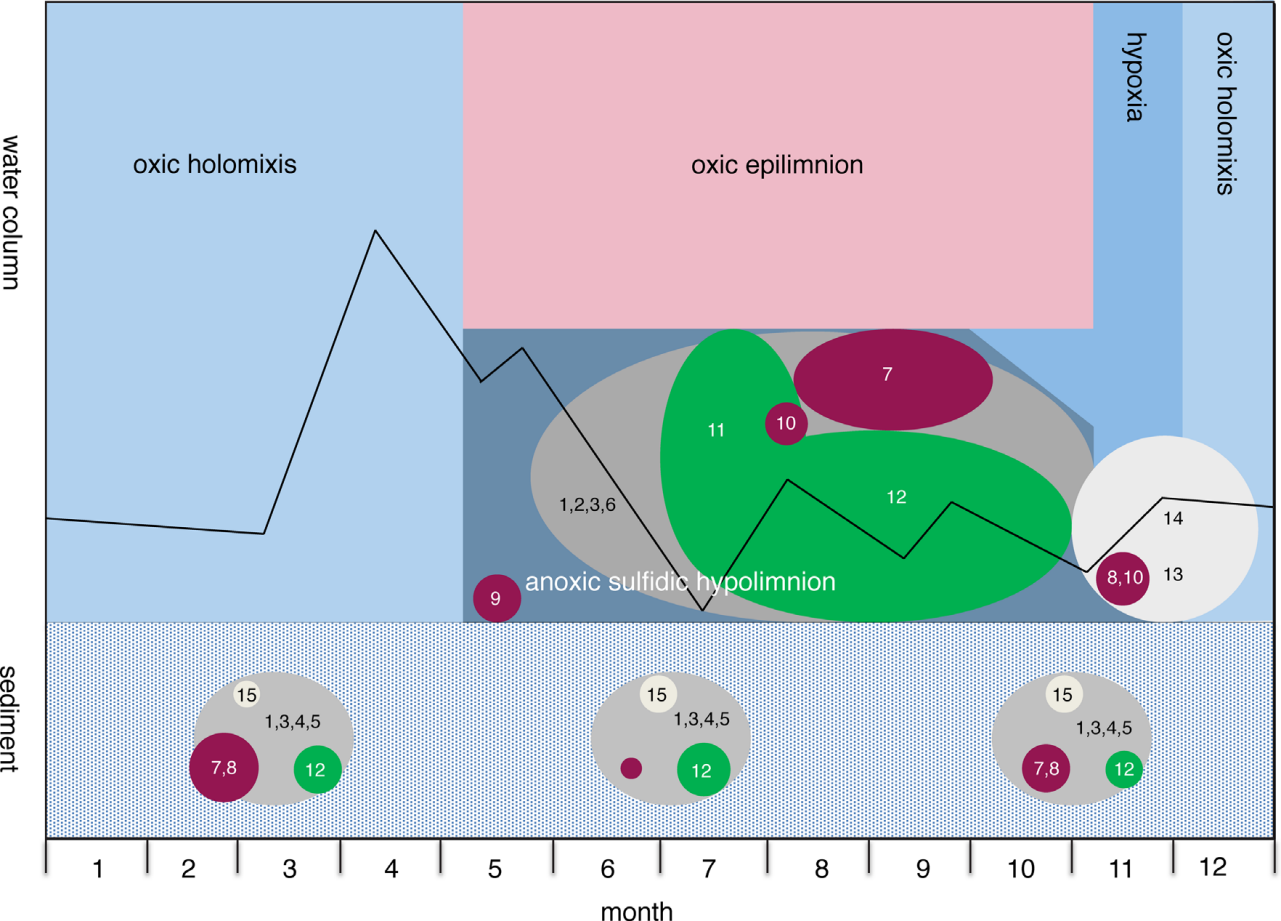
Conceptual model of the seasonal dynamics of sulfur bacteria in the water column and sediment of Lake Vechten. The model shows the seasonal stratification and oxygen conditions. Line indicates the bacterial euphotic depth. The circles represent the different functional groups (colors) and genera (numbers). Grey circles represent sulfate-reducing bacteria (1, *Desulfobulbus*; 2, *Desulfovibrio*; 3, *Desulfobacterium*; 4, unknown *Desulfobacteraceae*; 5, *Desulfuromonas*; 6, *Desulfomonile*), purple circles represent purple sulfur bacteria (7, *Thiodyction*; 8, *Lamprocystis*; 9, *Thiocystis*; 10, *Thiorhodococcus*), green circles represent green sulfur bacteria (11, unknown *Chlorobiaceae*; 12, *Chlorobium*) and white circles represent colorless sulfur bacteria (13, *Arcobacter*; 14, *Sulfurimonas*; 15, *Thiobacillus*). The size of the circles gives a rough indication of the relative abundances of the different bacteria.

Our observations and those from previous studies can be summarized in a simple conceptual model of the seasonal succession of sulfur bacteria in eutrophic lakes (Fig. 6). In winter and early spring, the water column is oxic and rich in sulfate. At this time, the sediment contains various sulfur bacteria including SRB using sulfate as terminal electron acceptor, and CSB and PSB oxidizing the upward flux of sulfide. After the water column stratifies in spring, oxygen and nitrate in the hypolimnion are depleted and subsequently a variety of SRB species expand into the anoxic hypolimnion, reducing sulfate to sulfide. During the summer period, photosynthetically active PSB bloom in the metalimnion and GSB thrive in the hypolimnion underneath, stimulated by high sulfide concentrations. When the lake is mixed during fall turnover, low oxygen concentrations are brought into the former hypolimnion, and SRB and GSB disappear from the water column because they are inhibited by oxygen. A combination of CSB and chemolithoautotrophic PSB flourish under these micro-aerobic conditions, oxidizing sulfide into sulfate. In winter, when all sulfide in the water column has been removed and oxic conditions in the water column are fully restored, sulfur bacteria disappear from the water column, but remain active in the sediment.

To the best of our knowledge, this is the first study that investigates the succession of SRB, GSB, PSB and CSB during oxic--anoxic transitions at a high taxonomic resolution (genus level) and summarizes the succession into a conceptual model. Admittedly, this simple conceptual model can still be improved in many ways. In particular, it will be an interesting challenge to assess in future studies which of these aspects are specific for Lake Vechten and which will apply to the seasonal succession of sulfur bacteria in other aquatic ecosystems. As discussed above, the similarities with other lake studies are promising (e.g. Noguerola *et al*. 2015; Pjevac *et al*. 2015).

Similarly, dynamics of nitrate and ammonium also showed seasonal patterns associated with the oxic--anoxic transitions (Fig. S1A and S1B, Supporting Information). A closer look at the 16S rRNA gene sequencing data revealed an increased relative abundance of denitrifiers during the first month of stratification in May (data not shown), when the hypolimnion became anoxic and nitrate concentrations in the hypolimnion were depleted. Furthermore, ammonium-oxidizing bacteria were detected in the sediment during fall turnover, accompanied by the accumulation of nitrate and decrease of ammonium. Meanwhile, methane-oxidizing bacteria (*Methylobacter*) flourished in the water column during fall turnover (see Fig. 5C in Diao *et al*. 2017), indicative of methane oxidation.

Recently, we showed that the interplay between microbial community dynamics and oxidation--reduction processes result in oxic--anoxic regime shifts characterized by tipping point and hysteresis effects (Bush *et al*. 2017). More specifically, degradation of organic matter by SRB creates anoxic and sulfidic conditions (also known as euxinia), and once ecosystems have become euxinic a large oxygen influx will be required to suppress the SRB, to overcome the low reduction potential that they have generated, and to bring the ecosystems back into their oxic state. Our results support and extend these findings. We observed that SRB were involved in the development of euxinia, that SRB remained abundant and euxinia was sustained throughout the summer period and that the hypolimnion did not return to its oxic state immediately after fall turnover. Instead, hypoxia developed throughout the water column in the fall, presumably because oxidation of the accumulated sulfide by CSB (e.g. *Arcobacter*) and chemolithotrophic PSB (e.g. *Lamprocystis*) consumed oxygen and thereby kept the DO concentrations low. Similarly, microbial oxidation of ammonium and other reduced compounds (e.g. methane) accumulated in the hypolimnion will also have consumed part of the oxygen influx. These results indicate that once ecosystems have become anoxic, the former oxic conditions are not easily restored.

## Supplementary Material

Supplementary material

## References

[bib2] Biderre-Petit C, Boucher D, Kuever J et al Identification of sulfur-cycle prokaryotes in a low-sulfate lake (Lake Pavin) using *aprA* and 16S rRNA gene markers. Microb Ecol. 2011;61:313–27. 21107833 10.1007/s00248-010-9769-4

[bib3] Biebl H, Pfennig N Growth yields of green sulfur bacteria in mixed cultures with sulfur and sulfate reducing bacteria. Arch Microbiol. 1978;117:9–16.

[bib4] Biggs R, Carpenter SR, Brock WA Turning back from the brink: detecting an impending regime shift in time to avert it. Proc Natl Acad Sci USA. 2009;106:826–31. 19124774 10.1073/pnas.0811729106 PMC2630060

[bib5] Bokulich NA, Rideout JR, Kopylova E et al A standardized, entensible framework for optimizing classification improves marker-gene taxonomic assignments. Peer J PrePrints. 2015;3:e934v2.

[bib6] Breitburg D, Levin LA, Oschlies A et al Declining oxygen in the global ocean and coastal waters. Science. 2018;359:eaam7240. 29301986 10.1126/science.aam7240

[bib7] Bush T, Diao M, Allen RJ et al Oxic-anoxic regime shifts mediated by feedbacks between biogeochemical processes and microbial community dynamics. Nat Commun. 2017;8:789. 28986518 10.1038/s41467-017-00912-x PMC5630580

[bib8] Camacho A, Vicente E, Miracle MR Spatio-temporal distribution and growth dynamics of phototrophic sulfur bacteria populations in the sulfide-rich Lake Arcas. Aquat Sci. 2000;62:334–49.

[bib9] Casamayor EO, García-Cantizano J, Pedrós-Alió C Carbon dioxide fixation in the dark by photosynthetic bacteria in sulfide-rich stratified lakes with oxic-anoxic interfaces. Limnol Oceanogr. 2008;53:1193–203.

[bib10] Chavez FP, Ryan J, Lluch-Cota SE et al From anchovies to sardines and back: multidecadal change in the Pacific Ocean. Science. 2003;299:217–21. 12522241 10.1126/science.1075880

[bib11] Cohen-Bazire G, Kunisawa R, Pfennig N Comparative study of the structure of gas vacuoles. J Bacteriol. 1969;100:1049–61. 4982667 10.1128/jb.100.2.1049-1061.1969 PMC250193

[bib12] Conley DJ, Carstensen J, Vaquer-Sunyer R et al Ecosystem thresholds with hypoxia. Hydrobiologia. 2009;629:21–9.

[bib13] Diao M, Sinnige R, Kalbitz K et al Succession of bacterial communities in a seasonally stratified lake with an anoxic and sulfidic hypolimnion. Front Microbiol. 2017;8:2511. 29312212 10.3389/fmicb.2017.02511 PMC5735980

[bib14] Diaz RJ, Rosenberg R Marine benthic hypoxia: a review of its ecological effects and the behavioural responses of benthic macrofauna. Oceanography and Marine Biology: An Annual Review. 1995;33:245–303.

[bib15] Diaz RJ, Rosenberg R Spreading dead zones and consequences for marine ecosystems. Science. 2008;321:926–9. 18703733 10.1126/science.1156401

[bib16] Edgar RC Search and clustering orders of magnitude faster than BLAST. Bioinformatics. 2010;26:2460–1. 20709691 10.1093/bioinformatics/btq461

[bib17] Edgar RC, Haas BJ, Clemente JC et al UCHIME improves sensitivity and speed of chimera detection. Bioinformatics. 2011;27:2194–200. 21700674 10.1093/bioinformatics/btr381 PMC3150044

[bib18] Edgar RC UPARSE: highly accurate OTU sequences from microbial amplicon reads. Nat Methods. 2013;10:996–8. 23955772 10.1038/nmeth.2604

[bib19] Edwardson CF, Hollibaugh JT Metatranscriptomic analysis of prokaryotic communities active in sulfur and arsenic cycling in Mono Lake, California, USA. ISME J. 2017;11:2195–208. 28548659 10.1038/ismej.2017.80 PMC5607362

[bib20] Eiler A, Heinrich F, Bertilsson S Coherent dynamics and association networks among lake bacterioplankton taxa. ISME J. 2012;6:330–42. 21881616 10.1038/ismej.2011.113 PMC3260505

[bib21] Eisenmann E, Beuerle J, Sulger K et al Lithotrophic growth of *Sulfurospirillum deleyianum* with sulfide as electron donor coupled to respiratory reduction of nitrate to ammonia. Arch Microbiol. 1995;164:180–5.

[bib22] Fox J, Weisberg S An R Companion to Applied Regression. SAGE Publications, 2011.

[bib23] Frigaard NU, Dahl C Sulfur metabolism in phototrophic sulfur bacteria. Adv Microb Physiol. 2009;54:103–200. 18929068 10.1016/S0065-2911(08)00002-7

[bib24] Ghosh W, Dam B Biochemistry and molecular biology of lithotrophic sulfur oxidation by taxonomically and ecologically diverse bacteria and archaea. FEMS Microbiol Rev. 2009;33:999–1043. 19645821 10.1111/j.1574-6976.2009.00187.x

[bib25] Gregersen LH, Bryant DA, Frigaard NU Mechanisms and evolution of oxidative sulfur metabolism in green sulfur bacteria. Front Microbiol. 2011;2:116. 21833341 10.3389/fmicb.2011.00116 PMC3153061

[bib26] Guerrero R, Montesinos E, Pedrós-Alió C et al Phototrophic sulfur bacteria in two Spanish lakes: vertical distribution and limiting factors. Limnol Oceanogr. 1985;30:919–31.

[bib27] Guyoneaud R, Caumette P, Imhoff JF Thiorhodococcus. Bergey's Manual of Systematics of Archaea and Bacteria. 2015, 1–7.

[bib28] Hamilton TL, Bovee RJ, Thiel V et al Coupled reductive and oxidative sulfur cycling in the phototrophic plate of a meromictic lake. Geobiology. 2014;12:451–68. 24976102 10.1111/gbi.12092

[bib29] Herlemann DPR, Labrenz M, Jürgens K et al Transitions in bacterial communities along the 2000 km salinity gradient of the Baltic Sea. ISME J. 2011;5:1571–9. 21472016 10.1038/ismej.2011.41 PMC3176514

[bib30] Imhoff JF Transfer of *Pfennigia purpurea* Tindall 1999 (*Amoebobacter purpureus* Eichler and Pfennig 1988) to the genus *Lamprocystis* as *Lamprocystis purpurea* comb. nov. Int J Syst Evol Microbiol. 2001;51:1699–701. 11594598 10.1099/00207713-51-5-1699

[bib31] Jenny JP, Francus P, Normandeau A et al Global spread of hypoxia in freshwater ecosystems during the last three centuries is caused by rising local human pressure. Glob Change Biol. 2016;22:1481–9. 10.1111/gcb.13193 26666217

[bib32] Kämpf C, Pfennig N Capacity of Chromatiaceae for chemotrophic growth. Specific respiration rates of *Thiocystis violacea* and *Chromatium vinosum*. Arch Microbiol. 1980;127:125–35.

[bib33] Kondo R, Osawa K, Mochizuki L et al Abundance and diversity of sulphate-reducing bacterioplankton in Lake Suigetsu, a meromictic lake in Fukui, Japan. Plankton Benthos Res. 2006;1:165–77.

[bib34] Kubo K, Kojima H, Fukui M Vertical distribution of major sulfate-reducing bacteria in a shallow eutrophic meromictic lake. Syst Appl Microbiol. 2014;37:510–9. 25034383 10.1016/j.syapm.2014.05.008

[bib35] Lennon JT, Jones SE Microbial seed banks: the ecological and evolutionary implications of dormancy. Nat Rev Microbiol. 2011;9:119–30. 21233850 10.1038/nrmicro2504

[bib36] Llorens-Marès T, Liu Z, Allen LZ et al Speciation and ecological success in dimly lit waters: horizontal gene transfer in a green sulfur bacteria bloom unveiled by metagenomic assembly. ISME J. 2017;11:201–11. 27392085 10.1038/ismej.2016.93 PMC5315485

[bib37] Manske AK, Glaeser J, Kuypers MMM et al Physiology and phylogeny of green sulfur bacteria forming a monospecific phototrophic assemblage at a depth of 100 meters in the Black Sea. Appl Environ Microbiol. 2005;71:8049–60. 16332785 10.1128/AEM.71.12.8049-8060.2005 PMC1317439

[bib39] Middelburg JJ, Levin LA Coastal hypoxia and sediment biogeochemistry. Biogeosciences. 2009;6:1273–93.

[bib40] Mori Y, Kataoka T, Okamura T et al Dominance of green sulfur bacteria in the chemocline of the meromictic Lake Suigetsu, Japan, as revealed by dissimilatory sulfite reductase gene analysis. Arch Microbiol. 2013;195:303–12. 23455488 10.1007/s00203-013-0879-5

[bib41] Muyzer G, Stams AJM The ecology and biotechnology of sulphate-reducing bacteria. Nat Rev Microbiol. 2008;6:441–54. 18461075 10.1038/nrmicro1892

[bib42] Muyzer G, Kuenen JG, Robertson LA Colorless sulfur bacteria. In: Rosenberg E et al (eds). The Prokaryotes - Prokaryotic physiology and biochemistry. Berlin: Springer, 2013, 555–88.

[bib43] Noguerola I, Picazo A, Llirós M et al Diversity of freshwater *Epsilonproteobacteria* and dark inorganic carbon fixation in the sulphidic redoxcline of a meromictic karstic lake. FEMS Microbiol Ecol. 2015;91:1–15. 10.1093/femsec/fiv086 26195601

[bib44] Oksanen J, Blanchet FG, Kindt R et al Vegan: Community Ecology Package. R package version 2.0-10 2013.

[bib45] Overmann J, Cypionka H, Pfennig N An extremely low-light-adapted phototrophic sulfur bacterium from the Black Sea. Limnol Oceanogr. 1992;37:150–5.

[bib46] Peduzzi S, Welsh A, Demarta A et al *Thiocystis chemoclinalis* sp. nov. and *Thiocystis cadagnonensis* sp. nov., motile purple sulfur bacteria isolated from the chemocline of a meromictic lake. Int J Syst Evol Microbiol. 2011;61:1682–7. 20729307 10.1099/ijs.0.010397-0

[bib47] Pfennig N, Trüper HG Anoxygenic phototrophic bacteria. In: Staley JT, Bryant MP, Pfennig N, Holt JG (eds). Bergey's Manual of Systematic Bacteriology. Baltimore: Williams & Wilkins, 1989, 1635–709.

[bib48] Pjevac P, Korlević M, Berg JS et al Community shift from phototrophic to chemotrophic sulfide oxidation following anoxic holomixis in a stratified seawater lake. Appl Environ Microbiol. 2015;81:298–308. 25344237 10.1128/AEM.02435-14 PMC4272751

[bib49] Saarenheimo J, Aalto SL, Syväranta J et al Bacterial community response to changes in a tri-trophic cascade during a whole-lake fish manipulation. Ecology. 2016;97:684–93. 27197395 10.1890/15-1052

[bib50] Schaub BEM, van Gemerden H Simultaneous phototrophic and chemotrophic growth in the purple sulfur bacterium *Thiocapsa roseopersicina* M1. FEMS Microbiol Ecol. 1994;13:185–96.

[bib51] Scheffer M, Carpenter SR Catastrophic regime shifts in ecosystems: linking theory to observation. Trends Ecol Evol. 2003;18:648–56.

[bib52] Schlitzer R Interactive analysis and visualization of geoscience data with Ocean data view. Comput Geosci. 2002;28:1211–8.

[bib53] Sievert SM, Wieringa EBA, Wirsen CO et al Growth and mechanism of filamentous-sulfur formation by *Candidatus* Arcobacter sulfidicus in opposing oxygen-sulfide gradients. Environ Microbiol. 2007;9:271–6. 17227432 10.1111/j.1462-2920.2006.01156.x

[bib54] Sorokin DY, Tourova TP, Muyzer G Isolation and characterization of two novel alkalitolerant sulfidogens from a Thiopaq bioreactor, *Desulfonatronum alkalitolerans* sp. nov., and *Sulfurospirillum alkalitolerans* sp. nov. Extremophiles. 2013;17:535–43. 23564266 10.1007/s00792-013-0538-4

[bib55] Steenbergen CLM, Korthals HJ Distribution of phototrophic microorganisms in the anaerobic and microaerophilic strata of Lake Vechten (The Netherlands). Pigment analysis and role in primary production. Limnol Oceanogr. 1982;27:883–95.

[bib56] Steenbergen CLM, Verdouw H Lake Vechten: aspects of its morphometry, climate, hydrology and physico-chemical characteristics. Hydrobiologia. 1982;95:11–23.

[bib57] Sweerts JRA, De Beer D, Nielsen LP et al Denitrification by sulphur oxidizing *Beggiatoa* spp. mats on freshwater sediments. Nature. 1990;344:762–3.

[bib58] Tonolla M, Peduzzi S, Hahn D et al Spatio-temporal distribution of phototrophic sulfur bacteria in the chemocline of meromictic Lake Cadagno (Switzerland). FEMS Microbiol Ecol. 2003;43:89–98. 19719699 10.1111/j.1574-6941.2003.tb01048.x

[bib59] Trüper HG, Schlegel HG Sulphur metabolism in Thiorhodaceae I. Quantitative measurements on growing cells of *Chromatium Okenii*. Antonie Van Leeuwenhoek. 1964;30:225–38. 14218435 10.1007/BF02046728

[bib60] Vaquer-Sunyer R, Duarte CM Thresholds of hypoxia for marine biodiversity. Proc Natl Acad Sci USA. 2008;105:15452–7. 18824689 10.1073/pnas.0803833105 PMC2556360

[bib61] Vila X, Abella CA Effects of light quality on the physiology and the ecology of planktonic green sulfur bacteria in lakes. Photosynth Res. 1994;41:53–65. 24310013 10.1007/BF02184145

[bib62] Walsby AE Gas vesicles. Microbiol Rev. 1994;58:94–144. 8177173 10.1128/mr.58.1.94-144.1994 PMC372955

[bib63] Yang J, Jiang HC, Dong HL et al Abundance and diversity of sulfur-oxidizing bacteria along a salinity gradient in four Qinghai-Tibetan lakes, China. Geomicrobiol J. 2013;30:851–60.

[bib64] Zhang J, Gilbert D, Gooday AJ et al Natural and human-induced hypoxia and consequences for coastal areas: synthesis and future development. Biogeosciences. 2010;7:1443–67.

[bib65] Zhang J, Kobert K, Flouri T et al PEAR: a fast and accurate Illumina Paired-End reAd mergeR. Bioinformatics. 2014;30:614–20. 24142950 10.1093/bioinformatics/btt593 PMC3933873

[bib66] Zuur A, Ieno EN, Walker N et al Mixed Effects Models and Extensions in Ecology with R, Berlin: Springer, 2009.

