# Supplementary material for: Spatio-temporal dynamics of sulfur bacteria during oxic--anoxic regime shifts in a seasonally stratified lake
Source: FEMS Microbiol Ecol. 2018 Mar 8;94(4):fiy040. doi: 10.1093/femsec/fiy040 (PMC5939864; doi:10.1093/femsec/fiy040)
Supplement: Supplementary material [file fiy040_supp.docx]

***Supplementary information***

# Spatio-temporal dynamics of sulfur bacteria during oxic-anoxic regime shifts in a seasonally stratified lake

Muhe Diao, Jef Huisman, Gerard Muyzer^§^

*Department of Freshwater and Marine Ecology, Institute for Biodiversity and Ecosystem Dynamics, University of Amsterdam, 1090 GE Amsterdam, The Netherlands*

*^§^Corresponding author: Gerard Muyzer -* [*g.muijzer@uva.nl*](mailto:g.muijzer@uva.nl)

***
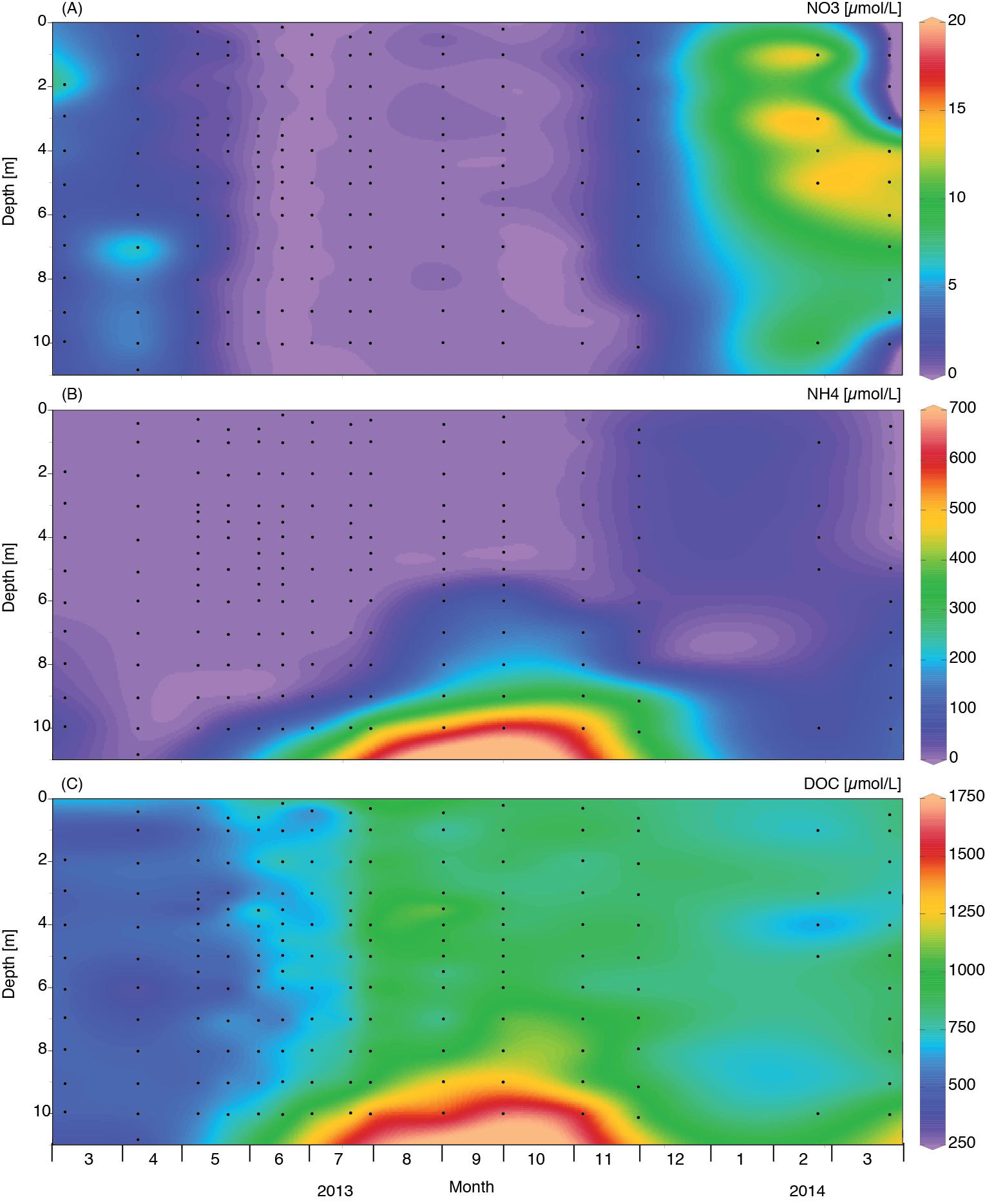
***

**Figure S1** Spatio-temporal dynamics of nutrients in Lake Vechten. (A) Nitrate; (B) ammonium; (C) dissolved organic carbon.

***
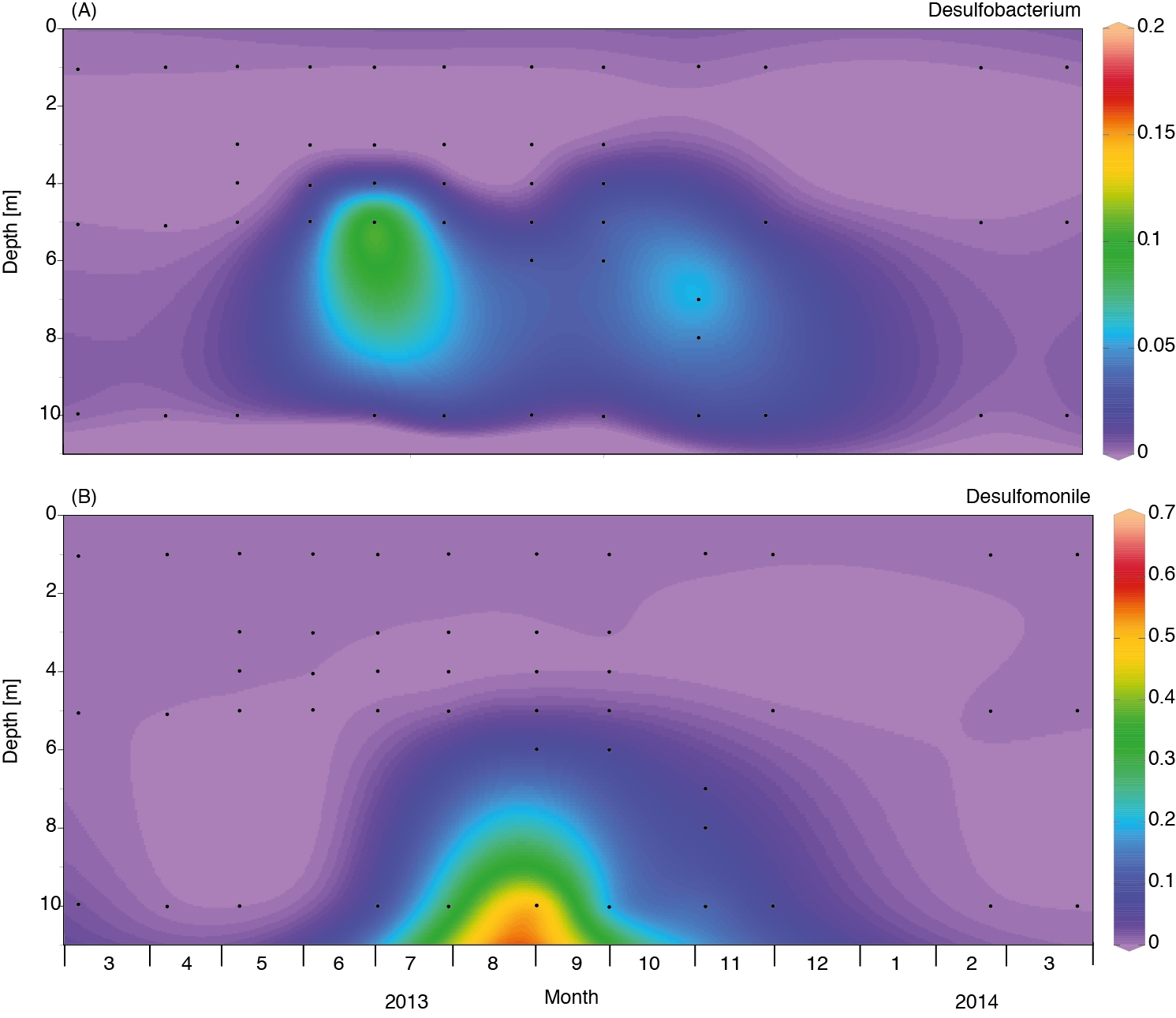
***

**Figure S2** Spatio-temporal dynamics of sulfate-reducing bacteria based on 16S rRNA gene amplicon sequences. The graphs show relative abundances (%) and distributions of (A) *Desulfobacterium*; (B) *Desulfomonile*.

***
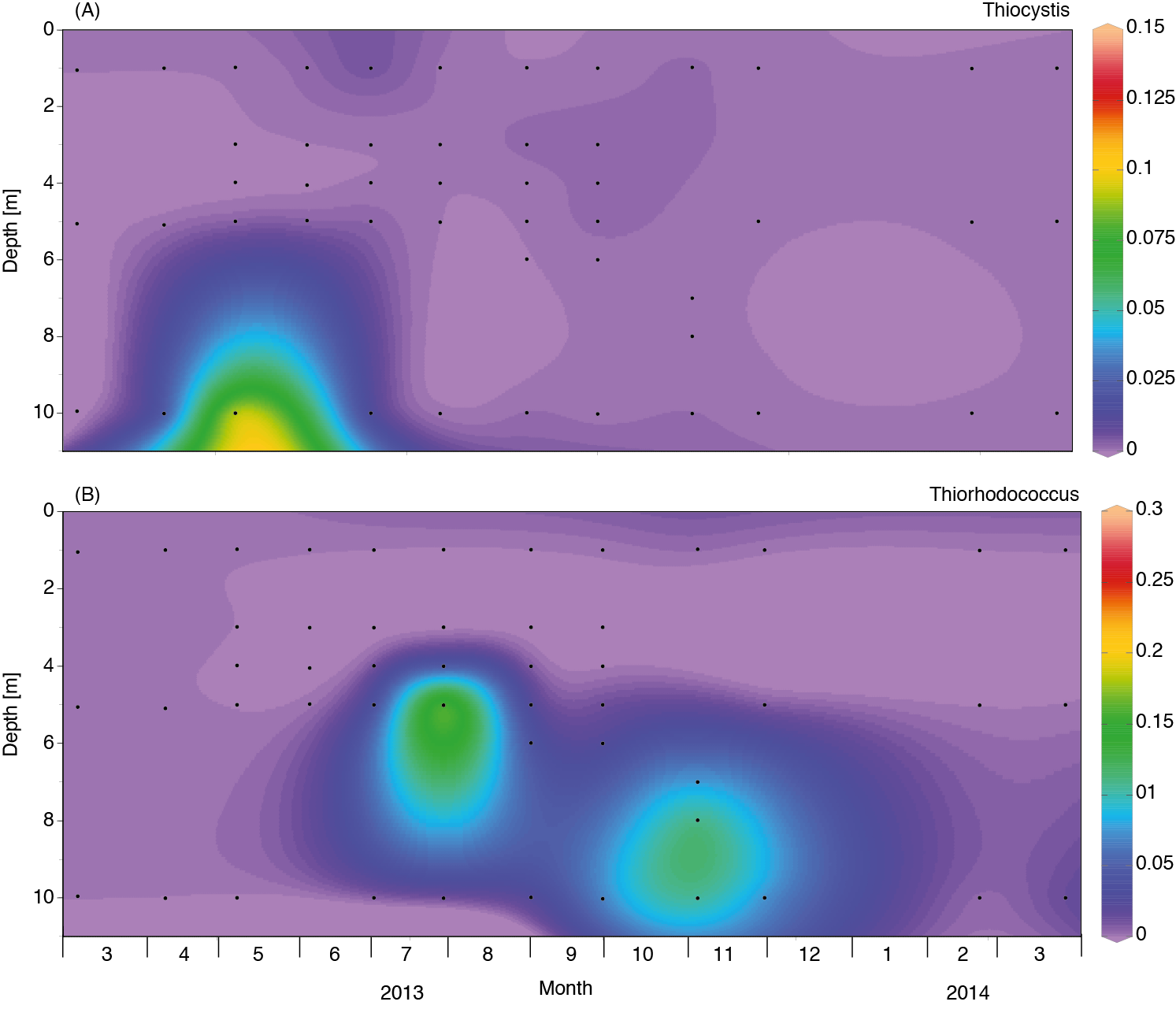
***

**Figure S3** Spatio-temporal dynamics of purple sulfur bacteria based on 16S rRNA gene amplicon sequences. The graphs show relative abundances (%) and distributions of (A) *Thiocystis*; (B) *Thiorhodococcus*.

**Table S1**. List of DNA samples for 16S rRNA gene amplicon sequencing

| **Sample ID** | **Date** | **Depth (m)** |  | **Sample ID** | **Date** | **Depth (m)** |
| --- | --- | --- | --- | --- | --- | --- |
|  |  |  |  |  |  |  |
| A0301 | 2013-03-06 | 1 |  | G0901 | 2013-09-02 | 1 |
| A0305 |  | 5 |  | G0903 |  | 3 |
| A0310 |  | 10 |  | G0904 |  | 4 |
| A03S |  | Sediment |  | G0905 |  | 5 |
| B0401 | 2013-04-10 | 1 |  | G0906 |  | 6 |
| B0405 |  | 5 |  | G0910 |  | 10 |
| B0410 |  | 10 |  | H0901 | 2013-09-30 | 1 |
| C0501 | 2013-05-08 | 1 |  | H0903 |  | 3 |
| C0503 |  | 3 |  | H0904 |  | 4 |
| C0504 |  | 4 |  | H0905 |  | 5 |
| C0505 |  | 5 |  | H0906 |  | 6 |
| C0510 |  | 10 |  | H0910 |  | 10 |
| D0601 | 2013-06-06 | 1 |  | I1101 | 2013-11-07 | 1 |
| D0603 |  | 3 |  | I1107 |  | 7 |
| D0604 |  | 4 |  | I1108 |  | 8 |
| D0605 |  | 5 |  | I1110 |  | 10 |
| D0610 |  | 10 |  | I11S |  | Sediment |
| E0701 | 2013-07-01 | 1 |  | J1201 | 2013-12-03 | 1 |
| E0703 |  | 3 |  | J1205 |  | 5 |
| E0704 |  | 4 |  | J1210 |  | 10 |
| E0705 |  | 5 |  | K0201 | 2014-02-25 | 1 |
| E0710 |  | 10 |  | K0205 |  | 5 |
| E07S |  | Sediment |  | K0210 |  | 10 |
| F0701 | 2013-07-29 | 1 |  | K02S |  | Sediment |
| F0703 |  | 3 |  | L0301 | 2014-03-31 | 1 |
| F0704 |  | 4 |  | L0305 |  | 5 |
| F0705 |  | 5 |  | L0310 |  | 10 |
| F0710 |  | 10 |  |  |  |  |

**Table S2** Significance of the selected explanatory variables in the RDA correlation triplots (see Fig. 5).

| **Explanatory Variable** | **AIC** | **Pseudo-F** | **P** |
| --- | --- | --- | --- |
| Sulfide | 100.34 | 17.46 | 0.005 |
| DO | 103.53 | 13.40 | 0.005 |
| SO_4_^2-^ | 107.03 | 9.23 | 0.005 |
| NH_4_^+^ | 107.83 | 8.32 | 0.005 |
|  |  |  |  |

*The explanatory variables were selected by forward selection based on the pseudo-F statistic, using 9999 permutations to assess their significance. AIC = Akaike information criterion. Total variation explained by the RDA model was 35.1%.*
